# Complete genomes of *Syntrophomonas wolfei* subsp. *saponavida* SD2^T^ and *Syntrophomonas wolfei* subsp. *methylbutyratica* 4J5^T^

**DOI:** 10.1128/mra.00531-26

**Published:** 2026-06-24

**Authors:** Ethan Humm, Jessica R. Sieber, Sung Min Ha, Neil Q. Wofford, Marco Morselli, Matteo Pellegrini, Michael J. McInerney, Robert P. Gunsalus

**Affiliations:** 1 Department of Microbiology, Immunology, and Molecular Genetics, University of California 8783, Los Angeles, California, USA; 2 Department of Biology, University of Minnesota-Duluth 14713, Duluth, Minnesota, USA; 3 Department of Integrative Biology and Physiology, University of California 8783, Los Angeles, California, USA; 4 Department of Microbiology and Plant Biology, University of Oklahoma 6187, Norman, Oklahoma, USA; 5 Department of Molecular, Cell, and Developmental Biology, University of California 8783, Los Angeles, California, USA; 6 UCLA DOE Institute, University of California 8783, Los Angeles, California, USA; University of Manitoba, Winnipeg, Manitoba, Canada

**Keywords:** *Syntrophomonas*, syntrophs, genomes, fatty acids

## Abstract

We report the genome sequences of two syntrophic bacteria, *Syntrophomonas wolfei* subsp. *saponavida* SD2^T^ and *Syntrophomonas wolfei* subsp. *methylbutyratica* 4J5^T^, that degrade a variety of short, branched, or long-chain saturated fatty acids to obtain energy when grown in co-culture with a suitable hydrogen-consuming microbial partner strain.

## ANNOUNCEMENT

Strains of the genus *Syntrophomonas* are members of the low-guanine-cytosine content (GC) syntrophic bacteria group within the family Syntrophomonadaceae (1, 2). *Syntrophomonas wolfei* subsp. *methylbutyratica* 4J5^T^ (=JCM 14075^T^) was isolated from rice field mud in a mineral medium with caproate and *Methanobacterium formicicum* DSM 1535^T^. The co-culture grows on C4–C8 linear chain fatty acids, 2-methyl butyrate, or beta-hydroxybutyrate (3). *Syntrophomonas wolfei* subsp. *saponavida* SD2^T^ (=DSM 4212^T^) was isolated from a sewage digestor in a mineral medium with tridecanoate and *Desulfovibrio* sp. G11. It grows on C4–C18 linear fatty acids when co-cultured with the hydrogenotrophic partner *Desulfovibrio* sp. G11 or *Methanospirillum hungatei* JF1^T^ (4). Both strains are gram-negative, variable straight to slightly curved rods that are motile by lateral flagella. These types of species are among the few described strains able to degrade substrates under thermodynamically limiting conditions, and their genome inventories provide insight into their metabolic potentials.

*Syntrophomonas wolfei* subsp. *saponavida* SD2^T^ and *S. wolfei* subsp. *methylbutyratica* 4J5^T^ were obtained from the McInerney laboratory culture collection and grown in pure culture on crotonate mineral salts medium anaerobically at 37°C as previously described (5). After 10 days of incubation, DNA was extracted using the Zymo D6060 Quick-DNA HMW Magbead Kit (Irvine, CA) per the manufacturer’s instructions and fragmented using Covaris gTubes following the manufacturer’s instructions (four passes at 7,000 rpm through the gTube orifice). The average size of the sheared gDNA was checked at the TapeStation 4200 (Agilent, Santa Clara, CA). Multiplexed microbial libraries were prepared using the PacBio SMRTbell prep kit 3.0 together with the SMRTbell barcoded adapters 3.0 according to the PacBio protocol (Menlo Park, CA). Final whole genome libraries were not size-selected but simply purified via a standard procedure using 1× SMRTbell clean-up beads. DNA sequencing was performed using the PacBio RSII platform. High-quality reads were assembled by Canu v2.2 (6), and the assembled genomes were further refined by Circlator v1.5.5 (7) to identify circular contigs and remove redundant non-circular contigs. A completeness check was performed by CheckM v1.0.18 (8) and the N50 quality was determined by Assembly stats v1.01 (https://github.com/sanger-pathogens/assembly-stats). Genome open reading frame (ORF) calling and annotation were performed by NCBI prokaryotic genome annotation pipeline (PGAP) v6.11 (9). Default parameters were used for all tools. The number of high-quality reads, completeness and N50 quality values are shown in Table 1 along with the properties of the *S. wolfei* subsp. *saponavida* SD2^T^ and *S. wolfei* subsp. *methylbutyratica* 4J5^T^ genomes. Genomic 16S gene sequence identity and average nucleotide identity (ANI) values were also calculated using the published sequences of the described type strains, respectively (3, 4), indicating close agreement.

**TABLE 1:** *S. wolfei* subsp. *saponavida* SD2T and *S. wolfei* subsp. *methylbutyratica* 4J5T genome sequence information

Strain	*S. wolfei* subsp. *saponavida* SD2ᵀ	*S. wolfei* subsp. *methylbutyratica* 4J5ᵀ
NCBI taxonomy ID	373074	378794
Culture collection	DSM 4212ᵀ	JCM 14075ᵀ
Topology	Circular	Circular
Size (bp)	3,590,077	3,227,150
GC% on isolation	ND^*a*^	47.3
GC% from genome	49.43	45.57
Protein coding genes	3,299	2,953
16S number	3	3
tRNA number	50	47
Coverage	33.6×	35.9×
Raw reads	59,361	41,865
Average read length	8,155 bp	5,486 bp
High-quality reads	12,259	8,936
Completeness	96.91%	98.72%
N50	3,590,077 bp	3,227,150 bp
16S identity	98.71–99.59%^*b*^	99.65–100%^*c*^
ANI	No genome available	99.99%^*d*^
Isolation source	Digestor sludge	Rice field mud
Location	Urbana, IL, USA	Jiangxi Province, China

^*a*^ ND, not determined.

^*b*^ genomic 16S to* S. wolfei* subsp. *saponavida* DSM 4212^T^ (acc. DQ666175.1).

^*c*^ genomic 16S to* S. wolfei* subsp.* methylbutyratica* 4J5^T^ (acc. NR_115800.1).

^*d*^ ANI to *S. wolfei* subsp. *methylbutyratica* JCM 14075^T^ (acc. GCA_001570625.1) (10).

The genomes of *S. wolfei* subsp. *saponavida* SD2^T^ and *S. wolfei* subsp. *methylbutyratica* 4J5^T^ each contain multiple paralogs of the beta-oxidation pathway enzymes for fatty acid activation and oxidation along with multiple gene loci for hydrogenases and formate dehydrogenases that allow for proton and electron disposal under thermodynamically limiting conditions (2). These syntrophic processes are ubiquitous in natural and man-made habitats and drive anaerobic carbon cycling in marine as well as freshwater environs (1, 2, 11).

## Data Availability

The *S. wolfei* subsp. *saponavida* SD2^T^ raw sequencing reads have been deposited under SRA accession number SRR35896236, and the assembled genome is listed under the GenBank accession number GCA_056726125.1. The *S. wolfei* subsp. *methylbutyratica* 4J5^T^ raw sequencing reads have been deposited under SRA accession number SRR35975359, and the assembled genome is listed under GenBank accession number GCA_056726105.1. The *S. wolfei* subsp. *saponavida* SD2^T^ IMG genome OID number is 8099421811 and the IMG submission ID is 338129 while the *S. wolfei* subsp. *methylbutyratica* 4J5^T^ IMG genome OID number is 8108582050 and the IMG submission ID is 338123.
